# Phase-Change Silicone Elastomers for Tough, Soft Actuators

**DOI:** 10.1021/acs.macromol.5c01221

**Published:** 2025-07-31

**Authors:** Yoo Jin Lee, Asaf Dana, Sasha M. George, Manivannan Sivaperuman Kalairaj, Yeh-Chia Tseng, Brandon M. Nitschke, Jared A. Gibson, Melissa A. Grunlan, Taylor H. Ware

**Affiliations:** † Department of Biomedical Engineering, 14736 Texas A&M University, College Station, Texas 77843, United States; ‡ Department of Materials Science and Engineering, Texas A&M University, College Station, Texas 77843, United States; § Department of Chemistry, Texas A&M University, College Station, Texas 77843, United States

## Abstract

Soft materials capable of controlled shape changes near ambient temperature are of interest for active devices that interact with living organisms. In this study, we achieve such functionalities by synthesizing responsive elastomers based on polydiethylsiloxane (PDES). Unlike conventional silicones, PDES elastomers are mesomorphic. Without any reinforcing additives, the mesophase improves the toughness of PDES to 8 times that of neat polydimethylsiloxane (PDMS) elastomers and 4 times that of Sylgard 184. Uniaxially stretched mesomorphic PDES elastomers undergo reversible shape changes under a bias load in response to temperature, generating 14% contractile strain on heating from 0 to 40 °C. The utility of PDES elastomers as actuators is enhanced by fabricating them into twisting actuators and describing strategies to minimize hysteresis during shape change cycles. The combination of toughness, actuation near ambient temperature, and environmental stability suggests that PDES could be attractive for biomedical devices where soft actuators interface with living organisms.

## Graphical abstract

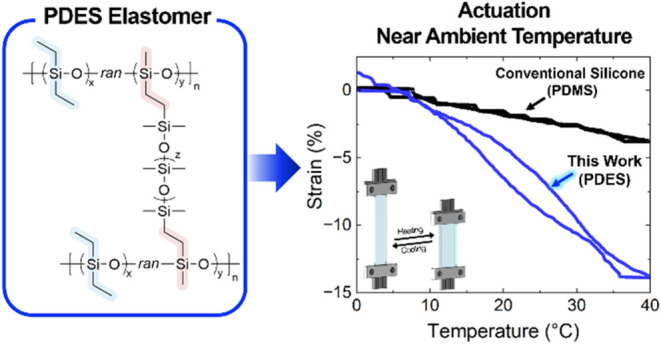

## 1 Introduction

Soft materials capable of reversible shape changes are of interest for devices that operate in dynamic environments. Biomedical devices,^1–4^ wearable devices,^5,6^ and soft robots^7,8^ often function under continuously changing environments, where soft, reversibly shape-changing materials can offer adaptability or perform tasks in response to environmental cues. A range of responsive materials have been developed that undergo programmed, reversible shape transformations in response to environmental changes. There are multiple mechanisms to create shape-changing polymeric materials, including hydrogel swelling/deswelling^9,10^ and entropic recovery of one-way shape memory polymers (SMPs).^11,12^ A principal mechanism for a reversible shape change without requiring mass transport involves polymers that undergo a phase transition between ordered and disordered phases. For example, two-way SMPs achieve reversible shape changes through the melting of crystal domains followed by crystallization on cooling.^13,14^ Liquid crystal elastomers (LCEs) undergo phase transition between ordered mesophases and disordered, isotropic phases to achieve large, reversible shape changes.^15–17^

Polymeric, reversibly shape-changing materials face several challenges that limit their broader application. Degradable shape-changing materials are suitable for short-term applications such as drug delivery,^18,19^ tissue scaffolds,^20,21^ and biodegradable robots.^22,23^ Few materials can achieve controlled, reversible shape transformations while maintaining mechanical integrity and functionality over extended periods of time in harsh environments. This limitation constrains their use in long-term applications, including implantable medical devices that can replace or augment human muscles^1,24,25^ and robots that operate under prolonged environmental stresses.^26^ In addition, many reversibly shape-changing materials exhibit low actuation stress and strain,^9,27^ along with significant reductions in the modulus upon actuation. In the case of thermally responsive materials, actuation often requires high temperatures,^27^ making it challenging to apply such materials for applications involving interactions with living organisms. Collectively, these challenges highlight the critical need to further develop soft, reversible, shape-changing materials to enable broader and more practical applications.

Mesomorphic silicone elastomers are promising candidates for soft, reversible shape-changing materials that address the key limitations of existing systems. The most commonly used silicones are elastomers based on poly­(dimethylsiloxane) (PDMS), but this amorphous material is relatively unresponsive to changes in environmental conditions. In contrast, poly­(di-*n*-alkylsiloxanes) substituted with ethyl up to hexyl side chains form mesophases that are absent in PDMS and offer intriguing properties.^28–30^ The elastomers exhibit mesophases identified as conformationally disordered crystals (condis crystals).^29,31–34^ In particular, uniaxially stretched polydiethylsiloxane (PDES) elastomers function as thermally responsive actuators as they undergo phase transitions between the condis crystal mesophase and the isotropic phase.^31,35–37^ Although the shape changes of PDES are reminiscent of those observed in LCEs, as both rely on phase transitions from ordered to disordered states to drive actuation, PDES and LCEs are fundamentally different. While the mesophase of LCEs stems from rigid, rodlike molecular segments,^38^ the condis crystal mesophase is found in polymers with high chain flexibility.^29,32,34,39^ Another key advantage of PDES elastomers lies in the chemical stability of the silicone elastomers. The chemical inertness of silicone elastomers ensures stability in vivo,^40,41^ supporting their potential uses in long-term applications.

Silicones exhibiting mesophases were actively studied until the early 2000s, with particular interest in PDES. Linear PDES with a molecular weight above approximately 30 kg mol^–1^ forms mesophases in addition to crystalline and amorphous phases.^42–44^ Cross-linked PDES elastomers are capable of forming similar phases. Notably, in addition to temperature-induced phase transitions, PDES elastomers can transition into the mesophase when stretched uniaxially. Once stretched beyond a critical ratio, PDES elastomers orient into an aligned mesophase.^35–37^ In the presence of a bias load that forms an aligned mesophase, PDES elastomers undergo a reversible shape change in response to temperature changes near room temperature.^37,45^

Several significant gaps in the research of PDES elastomers limit the current utility of these materials in engineering applications despite the demonstrated promise. For example, systematic control of material formulation and its effect on the actuation behavior is missing. Research on PDES has primarily focused on synthesizing high-molecular-weight PDES and understanding its fundamental physics. PDES elastomers were often prepared through peroxide vulcanization, yielding elastomers with a somewhat uncontrolled cross-link density.^46^ PDES elastomers were also synthesized by end-group cross-linking, allowing for better control over the cross-link density, but in these cases, the cross-link density was often fixed.^37^ Thus, systematic studies for tailoring PDES formulations to achieve a range of thermal and mechanical properties remain underdeveloped. Furthermore, despite the promise for actuators, the shape change of PDES elastomers has been limited to uniaxial strain, leaving more complex modes of actuation unexplored.

Here, we synthesize and characterize PDES elastomers with controlled thermal and mechanical properties (Figure 1a). Our approach for synthesizing PDES is suitable for making a range of elastomers and does not require an elaborate apparatus or extreme conditions. A systematic approach for synthesizing PDES elastomers with controlled cross-link densities is adapted from typical strategies to synthesize PDMS elastomers,^47,48^ thereby providing a tunable range of thermal and mechanical properties. It has been well established that materials exhibiting strain-induced phase transformations offer improved mechanical toughness.^49^ Similarly, PDES elastomers are found to outperform commercially available silicone products in terms of toughness. The PDES elastomers are fabricated with two distinct actuation modes: (1) contraction and expansion and (2) twisting and untwisting. Accelerated aging studies clearly indicate that PDES elastomers demonstrate enhanced resistance to both oxidation and hydrolysis compared to polyester LCEs and semicrystalline shape memory polymers. This combination of properties indicates promise for soft actuators requiring environmental stability.

**Figure 1:**
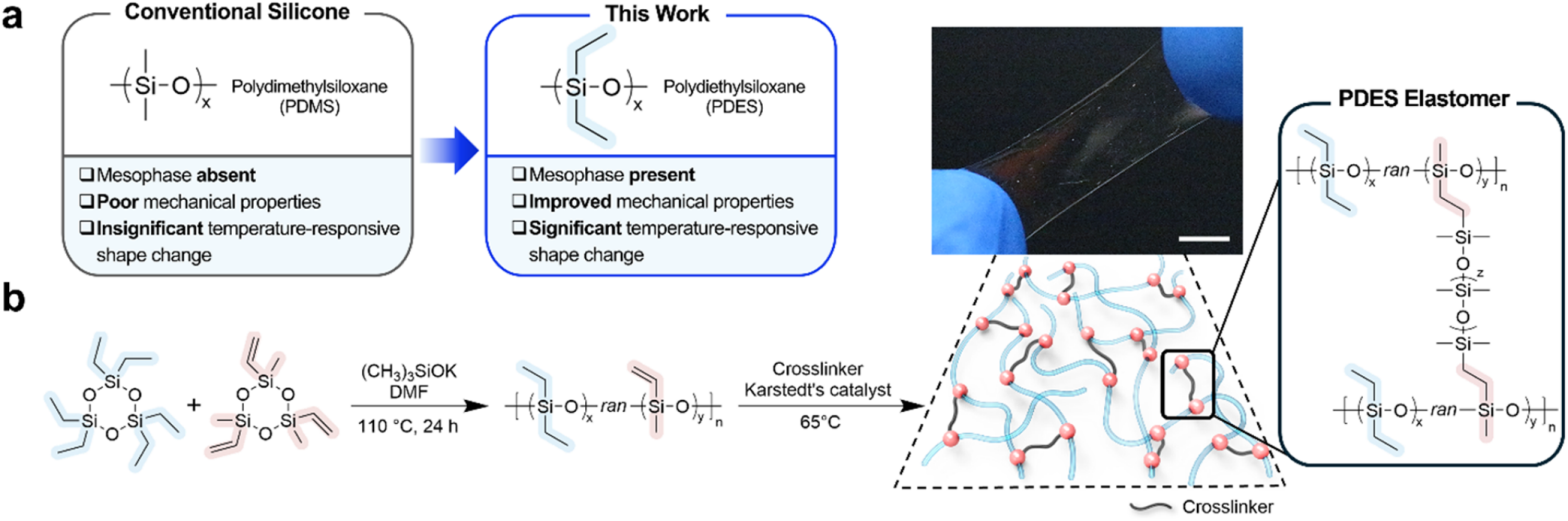
(a) Comparison of PDES-based silicone from this work to conventional PDMS-based silicone. (b) Synthesis of a linear PDES copolymer followed by cross-linking into a PDES elastomer. The resulting PDES elastomers are transparent elastomers, as shown in the picture with illustrated molecular structures. Scale bar: 5 mm.

## 2 Results and Discussion

### 2.1 Synthesis of PDES Copolymers

As a first step toward fabricating PDES elastomers, linear, vinyl-functionalized PDES copolymers were synthesized by anionic ring-opening polymerization of cyclic siloxanes (Figure 1b). The synthesis of linear PDES copolymers was performed by modifying a previously described method.^50^ The cyclic monomer, hexaethylcyclotrisiloxane (D_3_^Et^), was polymerized with (CH_3_)_3_SiOK as the initiator and *N*,*N*-dimethylformamide (DMF) as the promoter. A small amount of 1,3,5-trivinyl-1,3,5-trimethylcyclotrisiloxane (D_3_^Vi^) was added as a comonomer to synthesize linear PDES with vinyl side groups, which served as cross-link sites for preparing elastomers in the subsequent step. The molar ratio of D_3_^Et^ to D_3_^Vi^ was set at 98:2. This method of synthesis was chosen since it produced PDES of a moderately high molecular weight. The mesophase in PDES is only present when its molecular weight exceeds a critical threshold, which is about 30 kg mol^–1^.^42–44^ When the polymerization time was extended from 5 to 24 h at 110 °C, the number-average molecular weight of resultant PDES increased from 43 to 54 kg mol^–1^ (Figure 2a). While both reaction times resulted in PDES with a molecular weight over the critical threshold for forming mesophases, 24 h was selected as the polymerization time for this study as it consistently produced PDES around 54180 ± 671 g mol^–1^ with a yield of 70 ± 3% (*n* = 3).

**Figure 2:**
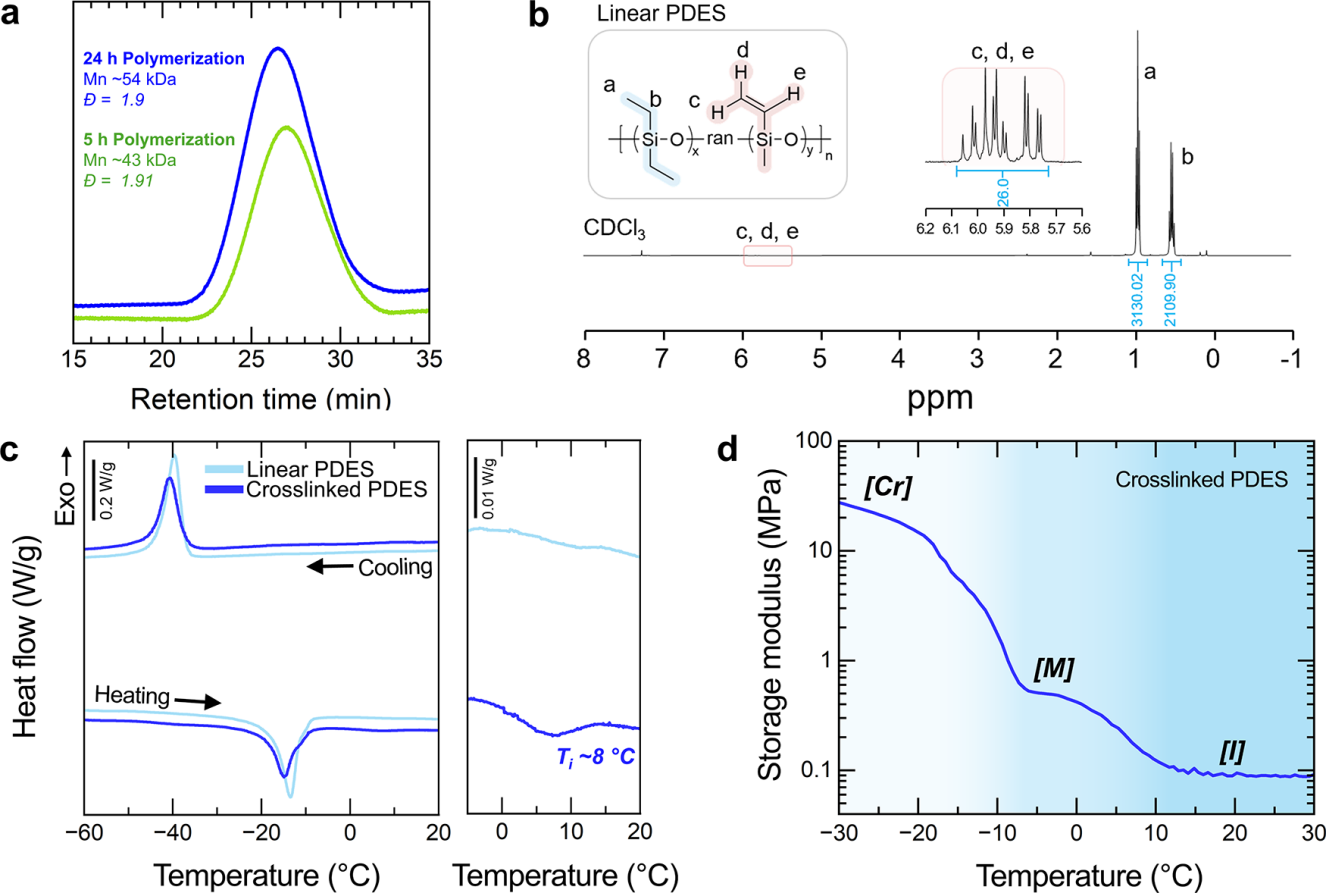
(a) GPC spectra of linear PDES copolymers with different polymerization times. Polymerization times of 5 and 24 h yield PDES copolymers with number-average molecular weights of 43 and 54 kg mol^–1^, respectively. (b) ^1^H NMR spectra of linear PDES copolymers in CDCl_3_, confirming the presence of ethyl and vinyl groups. (c) DSC spectra of linear and cross-linked PDES elastomers, with the image on the right providing a magnified view to highlight the presence of Ti. (d) Storage modulus of cross-linked PDES elastomers as a function of temperature. The phases corresponding to the crystalline phase ([Cr]), mesophase ([M]), and isotropic phase ([I]) are labeled. Consistent with DSC data, phase transitions are observed in similar temperature ranges.

The presence of vinyl groups on linear PDES copolymers was confirmed by ^1^H NMR spectroscopy. Two main sets of peaks were observed that correspond to protons from ethyl side groups (Figure 2b). Resonances corresponding to protons in −Si­(CH_2_C*H*_3_)_2_O– were clearly observed at 0.90–0.99 ppm (*a*) and 0.47–0.56 ppm (*b*). A small set of peaks detected at 5.8–6.1 ppm correspond to protons from vinyl side groups (*c,d,e*). The molar ratio of each repeat unit (D_3_^Et^/D_3_^Vi^) was calculated to be 98.3/1.7, yielding the average number of vinyl groups per polymer chain to be 8.9 ± 0.2 (*n* = 3). The calculated vinyl contents were used in subsequent steps to determine the amount of cross-linkers required for synthesizing elastomers at varying cross-link densities.

Linear PDES copolymers exhibit crystallinity and mesophases below room temperature. On heating, an overlap of two major peaks was observed between −20 and −8 °C by differential scanning calorimetry (DSC) (Figure 2c). PDES is reported to exist in two crystalline forms, referred to as α and β forms.^46,51,52^ The two peaks observed in DSC thermograms are attributed to crystal melting peaks of α and β forming into a mesophase. A peak corresponding to the melting of the mesophase into the isotropic phase was not clearly observed from the DSC analysis.

### 2.2 Synthesis of PDES Elastomers

Cross-linked PDES elastomers were prepared by hydrosilylation in the presence of a platinum catalyst. Vinyl groups from linear PDES copolymers and Si–H groups from hydride-terminated PDMS cross-linkers react at 65 °C to cross-link PDES copolymers into elastomers (Figure 1b), as confirmed by a gel fraction of 93.0 ± 2.0% (*n* = 3). PDES elastomers with a range of thermal, mechanical, and actuation properties were fabricated by varying the cross-link density. The cross-link density was controlled by adjusting the ratio of vinyl groups from linear PDES copolymers and Si–H groups from cross-linkers. The molar ratios of vinyl to Si–H groups were set at 100/40, 100/60, and 100/80, with the resulting elastomers referred to as 40, 60, and 80% cross-linked PDES elastomers, respectively. The presence of the mesophase in these PDES elastomers was confirmed by DSC (Figure 2c). In addition to two crystal melting peaks, the melting of the mesophase was clearly observed in 40% cross-linked PDES elastomers around 8 °C. The transition temperatures observed from DSC agree with the transition temperatures observed from dynamic mechanical analysis (Figure 2d). The storage modulus was measured as a function of temperature for 40% cross-linked PDES elastomers. The elastomers show an abrupt drop in the storage modulus at −20 °C, which corresponds to the crystal melt transition temperature of PDES elastomers. A smaller drop in the storage modulus is observed between 0 and 10 °C, which is the transition temperature from the mesophase to the isotropic phase. After the transition into the isotropic phase, PDES elastomers have a storage modulus of 92.7 ± 2.8 kPa at 20 °C (*n* = 3).

### 2.3 Mechanical Characterization

PDES elastomers with varying cross-link densities demonstrate different mesomorphic behaviors and mechanical properties. The Young’s modulus of PDES elastomers increases with the increasing cross-link density (Figure 3a, Table S1). As the cross-link density increases from 40 to 80%, the Young’s modulus increases from 113.3 ± 15.5 to 370.5 ± 26.1 kPa (*n* = 3). PDES elastomers have excellent toughness compared to PDMS elastomers with a comparable Young’s modulus (Figure 3b). As neat PDMS usually presents poor mechanical properties, commercially available PDMS products such as Sylgard 184 are engineered materials that contain reinforcing fillers such as silica.^53^ It is noteworthy that PDES elastomers without reinforcing fillers provide a mechanical performance similar to or surpassing that of engineered PDMS elastomers. The toughness of 80% cross-linked PDES was 8 times higher than that of neat PDMS and 4 times greater than that of Sylgard 184 with a comparable Young’s modulus. PDES elastomers with a lower cross-link density (60% cross-linked PDES) also demonstrate toughness 12 times higher than neat PDMS and 3 times higher than Sylgard 184 with a comparable Young’s modulus. PDES elastomers can exhibit high toughness up to 1.35 MJ m^–3^ without having reinforcing additives. We attribute the toughness of PDES elastomers to strain-induced mesophase formation.^54^ The impact of mesophases on improved mechanical properties has been observed in different mesomorphic materials such as poly­(lactic acid)^49,55^ and poly­(ethylene terephthalate).^56^ The strain-induced mesophase formation is also reminiscent of the improved toughness seen in natural rubber from strain-induced crystallization.^57^ PDES elastomers with varying cross-link densities provide a range of failure strengths in addition to the modulus and toughness (Figure 3c). The failure strength of PDES elastomers increases with the increasing cross-link density, while the failure strain remains nearly constant at 400%. The failure strain of PDES elastomers exceeds that of neat PDMS and Sylgard 184, while remaining below that of Ecoflex 00–30.

**Figure 3:**
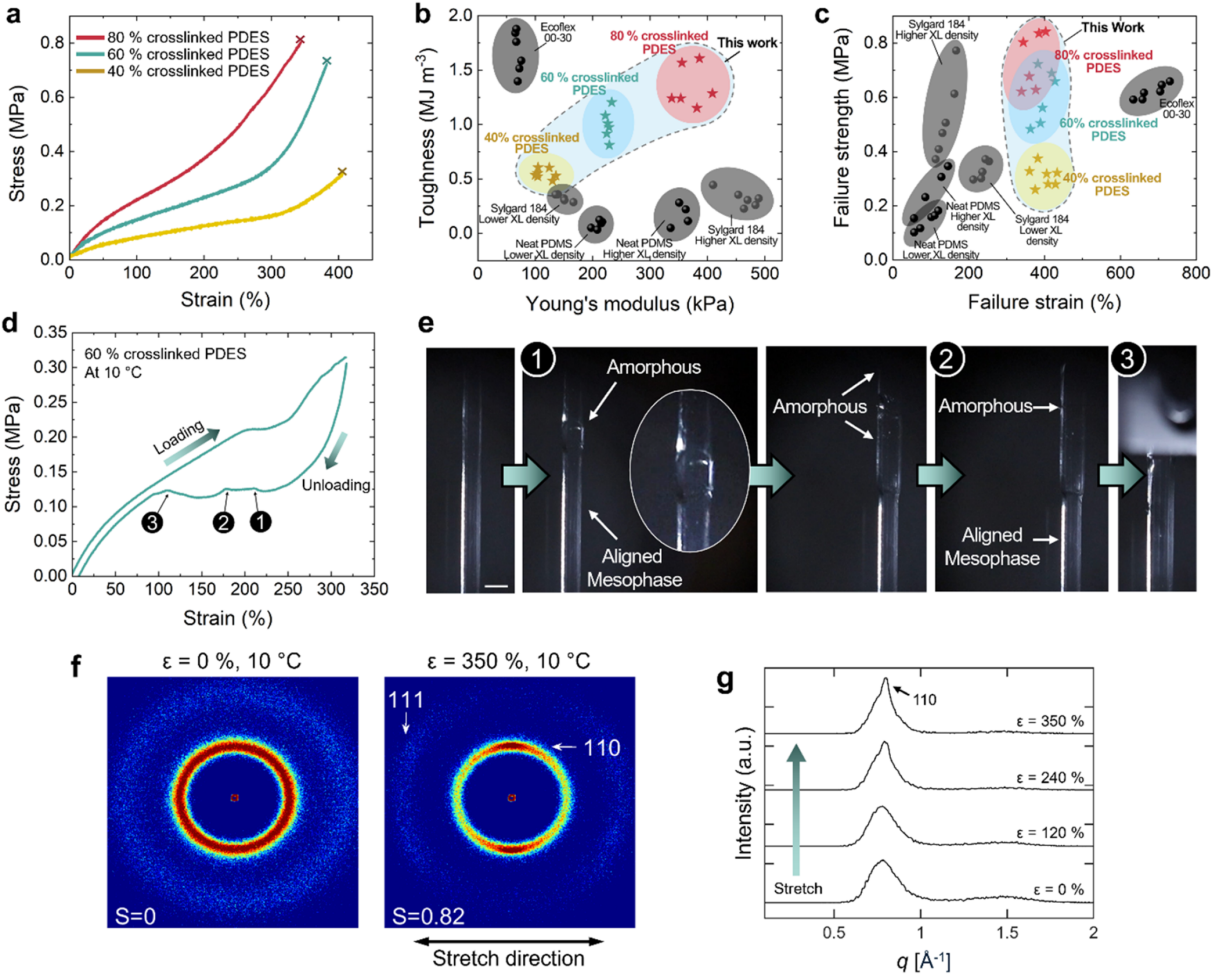
(a) Representative stress–strain curves from uniaxial tensile tests of PDES elastomers with varying cross-link densities, conducted at 20 °C. The failure point is marked on each curve. (b) Ashby plot of toughness versus Young’s modulus, highlighting the enhanced toughness of PDES elastomers compared to neat PDMS and Sylgard 184 at similar moduli. (c) Ashby plot of failure strength versus failure strain, demonstrating the mechanical performance across different formulations. (d) Representative stress–strain curves of PDES elastomers at 10 °C, revealing distinct plateaus during loading and unloading cycles. (e) Images taken at different stages of unloading at 10 °C, showing the initiation and propagation of the renecking process. Scale bar: 2 mm. (f) Wide-angle X-ray scattering (WAXS) patterns of PDES elastomers at 10 °C under different uniaxial strains. With the cross-link density fixed at 40%, unstretched PDES (left) exhibits isotropic patterns, which transform into anisotropic patterns under 350% stretch (right). (g) Change in the scattered intensity collected perpendicular to the direction of stretch as a function of the scattering vector with increasing strain at 10 °C.

The mechanical properties of PDES elastomers are significantly influenced by the temperature. To understand the influence of temperature on the mechanical properties, uniaxial cyclic loading and unloading tests were carried out on 60% cross-linked PDES elastomers at different temperatures (Figure S1). When the PDES elastomer was uniaxially stretched by 320% over its initial length at 10 °C, a small plateau was observed between 200 and 250% strain (Figure 3d). The appearance of the plateau is associated with the formation of the aligned mesophase under uniaxial tension.^32,35^ It has been reported that the plateau during the loading cycle indicates PDES transitioning from an amorphous to an aligned mesophase, accompanied by neck formation. In our studies, no necking was observed with increased loading, with elastomers homogeneously stretched across the sample. In contrast, the phase transition was clearly visible during the unloading cycle (Figure 3e, Video S1). While being unloaded, PDES elastomers transitioned from an aligned mesophase back to an amorphous phase. This transition created two distinct regions to appear in the sample, with narrower areas being in the aligned mesophase and wider areas being in the amorphous phase. During unloading, the amorphous regions emerge randomly across the sample with varying numbers of initiation points. The onset of the plateau in the stress–strain curve during unloading marks the beginning of this renecking, where wider amorphous regions emerge alongside the aligned mesophase. As unloading progresses, these amorphous regions expand until the entire sample transitions to the amorphous phase, marking the end of the plateau. Larger, more pronounced plateaus were observed at 0 °C. When the temperature was increased to 30 °C, the plateaus were not observed in either the loading or the unloading cycles. The appearance of the plateau due to strain-induced phase transition is not unique to the PDES elastomer; it is a common phenomenon across various material systems including shape memory alloys^58^ and natural rubbers, which undergo strain-induced crystallization.^59^ Additionally, the temperature had a significant impact on the hysteresis between loading and unloading cycles. As temperature increased, hysteresis decreased, becoming nearly absent at 30 °C (Figure S1). The effect of temperature on mechanical properties and hysteresis was also observed in 40% cross-linked PDES elastomers (Figure S2).

Mechanical stretching induces aligned mesophase formation in the PDES elastomers. In unstretched PDES elastomers, unaligned mesophases are present and melt around 8 °C (Figure 2c,2d). When PDES elastomers are mechanically stretched at 10 °C, stretching leads to an increase in the melting temperature of the mesophase and promotes the formation of aligned mesophases, as evidenced by Figure 3d–g. Under no strain (ε = 0%, 10 °C), PDES elastomers exhibited isotropic scattering patterns, indicative of the amorphous, isotropic phase (Figure 3f, left). Upon stretching (ε = 350%, 10 °C), scattering patterns became anisotropic, indicating the formation of an aligned mesophase. The phase transition was accompanied by an increase in the order parameter from approximately 0 to 0.82 (Figure S3a). The phase transition of PDES elastomers was strain-dependent. The effect of strain on phase transition was studied by measuring the scattered intensity along the meridian (perpendicular to the direction of stretch) as a function of the scattering vector, *q*, at varying strains (Figure 3g). As the strain increased, a sharp 110 peak emerged at *q* = 0.8 Å^–1^, corresponding to a *d* spacing of 7.85 Å, as opposed to the amorphous scattering with a *d* spacing of 8.16 Å. In addition, the amorphous scattering peak observed at *q* = 1.55 Å^–1^ in the unstretched sample (ε = 0%) disappeared with the increase in strain due to an increased anisotropy in the stretched sample. This change occurred since the 111 reflection (Figure 3f, right) appears only along azimuthal angles near the equatorial (Figure S4). The positions of the two anisotropic reflections are in strong agreement with the moduli of plane normals reported previously by several groups.^31,46^ WAXS results are consistent with results from uniaxial cyclic loading and unloading tests (Figure 3d), confirming that mechanical stretching induces phase transitions in PDES elastomers from the amorphous phase into the aligned mesophase.

### 2.4 Actuation Properties

Stretched PDES elastomers are thermally responsive actuators. Amorphous PDES elastomers were uniaxially stretched by 250% and cooled to 0 °C to generate the mesomorphic material (Figure 4a). The mesomorphic PDES elastomers were prepared in this way and maintained with a bias load contract uniaxially as the mesophase melts with increasing temperature. We first study the generation of stress in PDES elastomers, while maintaining a fixed length (isostrain). PDES elastomers with the highest cross-link density (80% cross-linked PDES) show the highest blocking stress of 0.52 ± 0.01 MPa on heating (*n* = 3). The magnitude of stress generated at a fixed strain of 250% decreased with an increasing cross-link density. Lower contractile stresses of 0.37 ± 0.03 and 0.21 ± 0.03 MPa were produced in 60 and 40% cross-linked PDES elastomers, respectively (*n* = 3). Control PDMS elastomers (Ecoflex 00–30) also undergo thermally reversible shape changes under a bias load as would be expected for any elastomer.^60^ However, amorphous PDMS only produces one tenth of the maximum actuation stress of 80% cross-linked PDES elastomers (contractile stresses: 0.05 ± 0.01 MPa) over the same temperature range. Repeated thermal cycling demonstrates the reversibility of the actuation behavior of PDES elastomers (Figure 4b). PDES elastomers were cycled 4 times between 0 and 40 °C, with the actuation stress remaining constant throughout the cycles.

**Figure 4:**
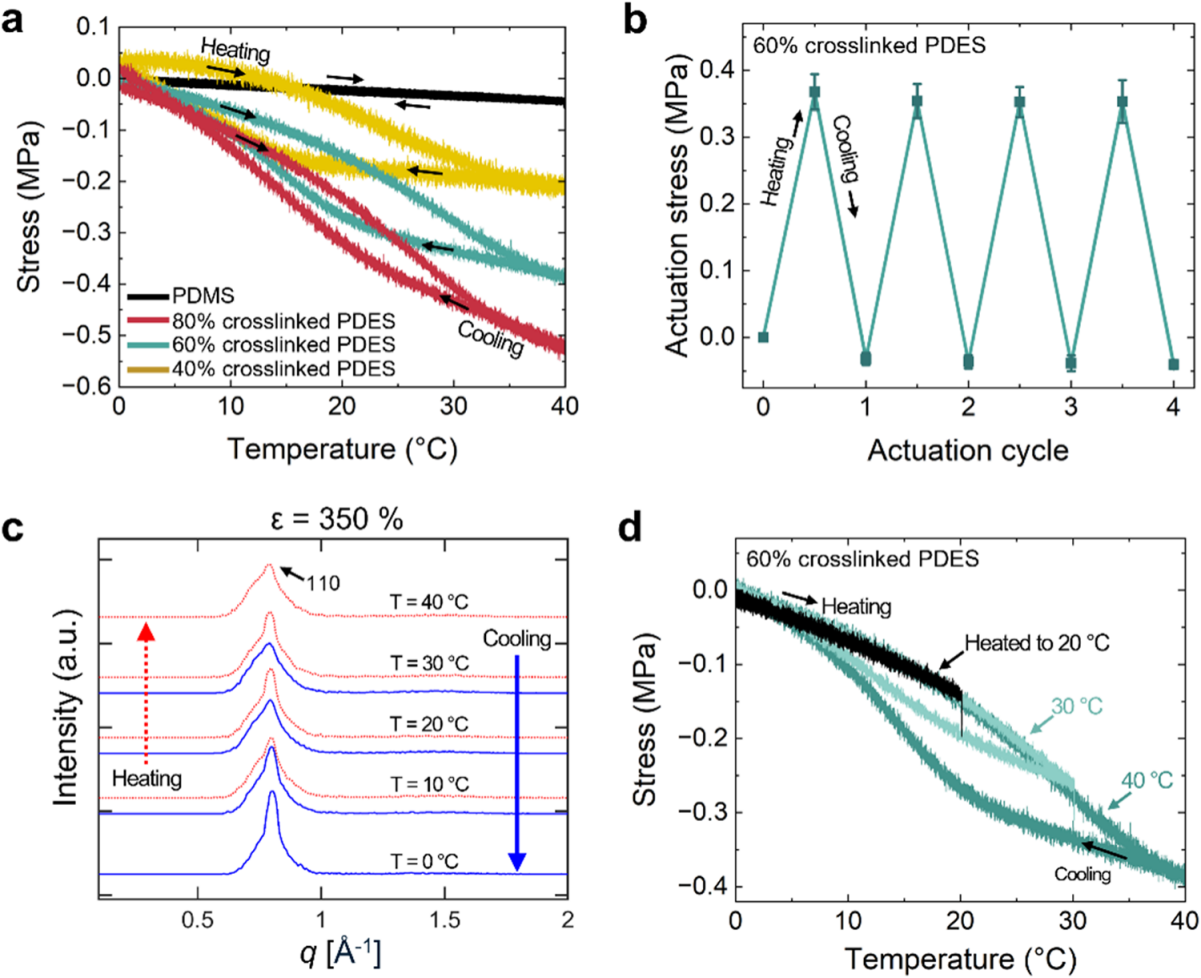
(a) Contractile stress generated by PDES elastomers with varying cross-link densities. The presence of a mesophase in PDES enhances contractile stress compared to PDMS. (b) Reversibility of actuation evaluated by cyclic testings, showing consistent actuation stress over four cycles. (c) Structural transition of PDES elastomers in response to temperature changes. WAXS was performed on 40% cross-linked PDES elastomers under a fixed uniaxial strain of 350%. The scattered intensity along the meridian was measured as a function of the scattering vector during heating and cooling to capture the temperature-dependent structural changes. (d) Hysteresis in actuation is controlled by partial melting of the mesophase, reducing hysteresis to near zero when heated only up to 20 °C.

The PDES elastomers synthesized in this study exhibit an actuation behavior comparable to previously reported systems, but there are important new insights. Important initial efforts to characterize PDES elastomers did not provide a systematic approach to control the cross-link density and used a variety of cross-linking mechanisms.^36,54^ In addition, the phase transition temperature was sometimes reported to be near 100 °C under large stresses. However, there are unclear aspects of these efforts, such as the molecular weight of PDES before cross-linking. Other elastomers prepared by cross-linking the end groups of linear, moderate-molecular-weight PDES provide sufficient details for direct comparison.^37^ PDES elastomers in our study demonstrate slightly broader actuation temperature ranges, but the phase transition temperatures are very similar. Cross-linking exclusively at the chain ends tends to produce more uniform polymer lengths between cross-links, which may lead to narrower actuation temperature ranges. We note that the prior report showed a higher actuation strain, but with a lower elastic modulus.^37^ Again, the focus of that important effort was not systematic control of the cross-link density. Here, tuning the cross-link density strongly influences the stress generated from actuation (Figure 4a). The effect of the cross-link density on the actuation behavior extends beyond the magnitude of stress generated. Hysteresis between heating and cooling cycles varies with the cross-link density, with 80% cross-linked PDES elastomers showing the lowest hysteresis, while lower cross-link densities lead to increased hysteresis. We cannot rule out the fact that the degree of mesophase formation is also affected by the cross-link density over this range.

The mesophase of PDES elastomers forms and melts in response to temperature changes. WAXS patterns were collected during the heating and cooling cycle of the PDES elastomer under a fixed strain of 350%. Upon heating from 10 to 40 °C, the intensity of the 110 peak decreased, indicating the melting of the aligned mesophase into the amorphous phase (Figures 4c, S3d). The melting of the aligned mesophase on heating is supported by the decrease in the order parameter (Figure S3c). When the PDES elastomer was heated 20 °C immediately after stretching at 10 °C, the 110 peak intensity slightly increased, possibly due to the somewhat slow kinetics of mesophase formation. During subsequent cooling, the 110 peak intensity increased again (Figure S3d), with the most pronounced intensity and alignment observed at 0 °C, confirming the reformation of the aligned mesophase (Figure 4c). Hysteresis was observed in both the 110 scattered intensity and the order parameter during the thermal cycle, aligning with the hysteresis observed in isostrain tests (Figures 4a and S3).

Hysteresis in actuation cycles of PDES elastomers can be controlled by partially melting the mesophases during heating cycles. PDES elastomers, regardless of the cross-link density, exhibit hysteresis during actuation cycles (Figure 4a). From a practical perspective, hysteresis between heating and cooling cycles complicates the use of a responsive material as an actuator. In semicrystalline shape memory polymers, hysteresis in actuation cycles can be controlled by partially melting crystalline regions during heating. In this approach, the remaining crystallites function as latent templates for crystallization.^14^ We hypothesized that the same approach can be used for controlling the inherent hysteresis observed in PDES elastomers. To validate the hypothesis, 60% cross-linked PDES elastomers were heated to different temperatures to vary the extent of mesophase melting (Figure 4d). When heated to 40 °C, where the mesophase was fully melted, the hysteresis between the heating and cooling cycles was the largest. In contrast, when the mesophase was partially melted at 20 °C, the elastomers exhibited almost no hysteresis. These results demonstrate effective control over the hysteresis of PDES elastomers through partial mesophase melting, highlighting their potential for actuators where controlling hysteresis is critical.

Unaxially stretched PDES elastomers undergo contraction upon heating and elongation upon cooling under a constant bias load (isostress). Contractile strain produced from 80% cross-linked PDES elastomers is 14% over a temperature window of 0 to 40 °C (Figure 5a) with a bias stress of 410 kPa. As expected, control PDMS elastomers (Ecoflex 00–30) show a much smaller contractile strain of 4% with no abrupt changes in the slope of the strain as a function of the temperature plot during isostrain tests. For 60 and 40% cross-linked elastomers, the prestretching stress was not high enough to be maintained throughout the test due to instrument limitations. Thus, isostrain results of 60 and 40% cross-linked PDES elastomers are not included.

**Figure 5:**
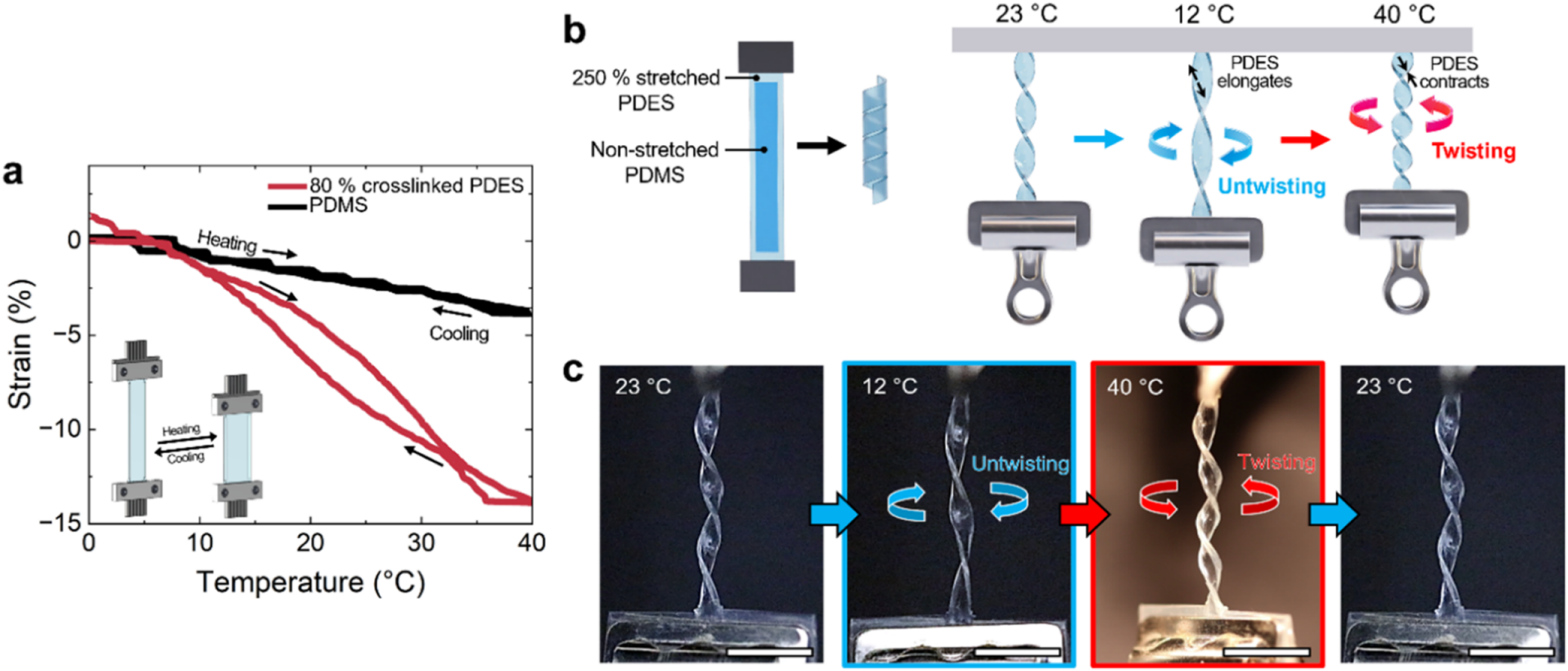
(a) Actuation strain of PDES elastomers under uniaxial stretching, undergoing contraction and elongation in response to temperature. The presence of a mesophase enhances strain compared with PDMS elastomers. (b) Schematic illustrating the fabrication process of twisting actuators and their thermally responsive twisting and untwisting behaviors. (c) Photographs capturing the thermally responsive actuation of twisting and untwisting actuators. Scale bar: 1 cm.

The utility of PDES elastomers can be expanded by fabricating the materials into twisting actuators. Twisting actuators were fabricated by building bilayer structures, where 250% stretched PDES elastomers serve as an active layer and unstretched PDMS elastomers function as a passive layer (Figure 5b). After fabrication, the bilayer-based actuator adopts a twisted form once the external load is removed. This twisted form is dictated by the amount of recovered strain and the aspect ratio of the bilayer, as has been observed previously.^61^ The bilayer-based actuator exhibits thermally responsive twisting and untwisting actuation driven by the phase transitions of the PDES elastomers. Upon cooling, the active layer of the PDES elastomer expands along the long axis. As a result, the length of PDES and PDMS becomes better matched, causing the actuator to untwist (Figure 5b,5c). On heating, the PDES elastomer contracts and drives the actuator to twist (Figure 5b,5c, Video S2). Since the thermally responsive shape changes of the PDES elastomer as the active layer are reversible, twisting and untwisting actuation of the bilayered actuators is also reversible in response to temperature.

### 2.5 Environmental Stability of PDES Elastomers

PDES elastomers are environmentally stable when exposed to accelerated hydrolytic and oxidative conditions. Silicones are known to have good environmental stability, which makes them desirable materials for applications, such as long-term implantable biomedical devices. PDES elastomers with a 60% cross-link density show 0 ± 0.2% and 0 ± 0.1% change in the mass after 15 days in accelerated hydrolytic and oxidative conditions, respectively (*n* = 3). (Figure 6a,b). These conditions are thought to represent over 6 to 7 months in vivo based on the comparison to studies on poly­(carbonate urethanes).^62^ The environmental stability of PDES elastomers was further validated by analyzing changes in the actuation performance before and after degradation (Figure 6c). There is no significant change in the blocking stress (*p* = 0.6279, one-way ANOVA) or temperature range of actuation after simulated degradation, demonstrating that PDES elastomers show good hydrolytic and oxidative stability.

**Figure 6:**
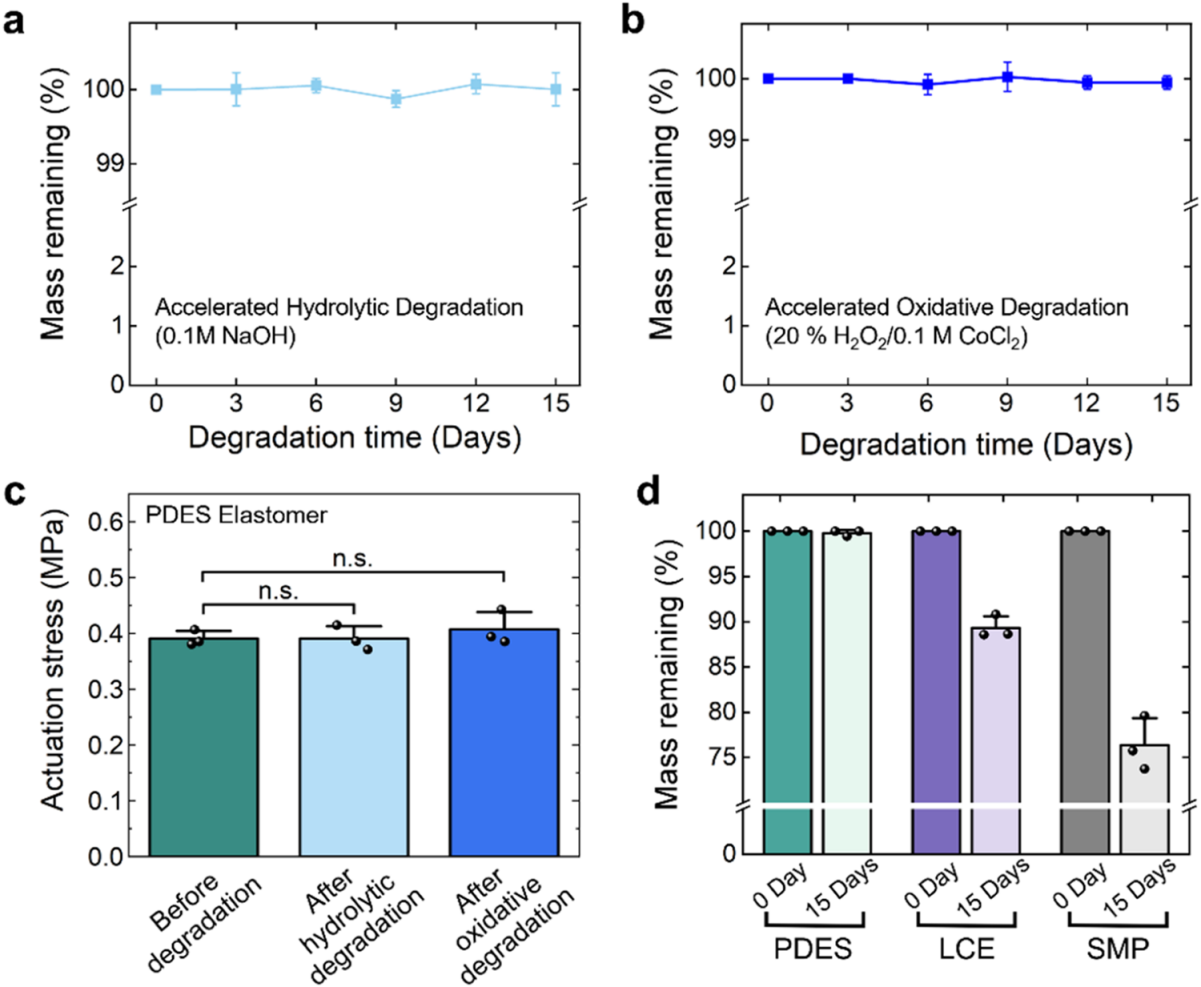
(a) Mass changes of PDES elastomers under accelerated hydrolytic degradation. (b) Mass changes under accelerated oxidative conditions. (c) Actuation stress remaining largely unchanged before and after degradation. (d) PDES elastomers exhibiting no mass loss, whereas LCEs and SMPs showing a decline after 15 days under accelerated oxidative conditions.

The environmental stability of PDES is notable compared to other types of shape-changing materials. Semicrystalline SMPs, which are capable of reversible shape change with a bias load and are often polyesters (e.g., cross-linked poly­(ε-caprolactone-diacrylate) (PCL)), are vulnerable to hydrolytic and oxidative conditions, making them suitable for biodegradable devices.^63^ While many LCEs provide some resistance to degradation in hydrolytic conditions due to the intrinsic hydrophobic nature, LCEs still degrade to varying degrees.^64,65^ In vitro degradation tests comparing the dry mass retention of representative SMPs, representative LCEs, and PDES elastomers confirm the excellent environmental stability of the PDES elastomers (Figure 6d). After 15 days under accelerated oxidative conditions, PDES elastomers retained 99.8 ± 0.3% of the initial mass, whereas both LCEs and SMPs exhibit a larger mass loss, with 89.3 ± 1.3 and 76.4 ± 3.0% mass remaining, respectively. The environmental stability of PDES elastomers offers advantages, particularly in biomedical applications where biostability is critical. PDES-based elastomers and actuators could be promising candidates for developing implantable medical devices where long-term biostability is essential, contributing to the device durability over extended periods.

## 3 Conclusions

There is a need to develop responsive materials that undergo controlled changes in form at temperatures near room temperature or physiological conditions that are environmentally stable. Here, we describe a formulation strategy for PDES elastomers, whose unique mesophases impart them with excellent toughness and shape-changing abilities near ambient temperature. These silicones lack any mesogen typically associated with liquid crystalline materials. We demonstrated approaches to systematically control the cross-link density, which controls the actuation stress and strain magnitude. While the shape change of these materials is hysteretic on heating and cooling, partial melting of the mesophase can be used to eliminate the hysteresis. The PDES elastomer temperature-responsive actuators are capable of contracting and elongating or twisting and untwisting with temperature change. PDES elastomers exhibited excellent environmental stability compared with other shape-changing materials. We envision PDES elastomers as promising candidates for a wide range of applications, particularly in biomedical applications.

## 4 Experimental Section

### 4.1 Materials

Hexaethylcyclotrisiloxane (D3^Et^; purity: 97%), 1,3,5-trivinyl-1,3,5-trimethylcyclotrisiloxane (D3^vi^; purity: 97%), the hydride-terminated poly­(dimethylsiloxane) cross-linker (1000–1100 g mol^–1^), and the linear PDMS copolymer (4–5 mol % vinylmethylsiloxane, 800–1,200 cSt) were purchased from Gelest, Inc. Potassium trimethylsilanolate ((CH_3_)_3_SiOK; purity: 96%) was purchased from Ambeed. *N*,*N*-Dimethylformamide (DMF; purity: 99.9%), toluene (anhydrous, purity: 99.8%), and chloroform were purchased from Fisher Scientific. Karstedt’s catalyst (∼2% of Pt in xylene) was purchased from Sigma-Aldrich. Glacial acetic acid (purity >99.7%) was purchased from Thermo Scientific Chemicals. Sodium hydroxide pellets (NaOH) were purchased from Fisher Chemical. 2,2-(Ethylenedioxy)­diethanethiol (EDDT), 1,3,5-triallyl-1,3,5-triazine-2,4,6­(1H,3H,5H)-trione (TATATO), Irgacure 369 (I-369), Irgacure 819 (I-819), butylated hydroxytoluene (BHT), triethylamine (TEA), cobalt chloride (CoCl_2_; purity: 97%), PCL-diol (*M*_n_–10 kg mol^–1^), 4-dimethylaminopyridine, triethylamine, acryloyl chloride, 2,2-dimethoxy-2-phenylacetophenone (DMP), and *N*-vinyl-2-pyrrolidone (NVP) were purchased from Sigma-Aldrich. The liquid crystal monomer 1,4-bis-[4-(6-acryloyloxyhexyloxy)­benzoyloxy]-2-methylbenzene (RM82) was purchased from Daken Chemical. The Sylgard 184 PDMS kit was purchased from Electron Microscopy Sciences. Ecoflex 00–30 and Ecoflex 00–35 Fast were purchased from Smooth-On. All chemicals used in this study were used as received without further purification.

### 4.2 Synthesis of Linear PDES Copolymers

A monomer solution of D3^Et^ (7 mL, 21.8 mmol) and D3^vi^ (120 μL, 0.449 mmol) was prepared and stirred. (CH_3_)_3_SiOK (1.3 mg, 10.1 μmol), DMF (30 μL, 0.387 mmol), and the monomer solution (3 mL) were added to a 25 mL round-bottom flask. The reaction mixture was stirred at room temperature for 1 min and then heated to 110 °C. The polymerization was carried out for 24 h under dry nitrogen. After the reaction time, acetic acid (350 mg) was added to quench the reaction. Toluene (8 mL) was added to the mixture to reduce the viscosity. After 10 min of stirring at room temperature, the mixture was transferred to a 50 mL centrifuge tube, and 30 mL of deionized water was added. The tube was centrifuged at 4000 rpm for 10 min at room temperature. After centrifugation, the top layer containing the polymer was collected. This process was repeated 4 times to neutralize the polymer chain ends and to remove the potassium salt/promoter complex. The polymer was then precipitated via centrifugation using methanol. This process was repeated 5 times to remove any unreacted monomers and oligomeric byproducts. The precipitated polymer was collected and then dried in a vacuum chamber at room temperature for 24 h.

### 4.3 Synthesis of PDES Elastomers

PDES elastomers were synthesized via the hydrosilylation of vinyl groups of linear PDES copolymers and Si–H of the cross-linker in the presence of a platinum catalyst. In a 5 mL vial, the linear PDES copolymer, the cross-linker, Karstedt’s catalyst, and toluene were added and stirred using a Thinky Mixer for 2 min. Both Karstedt’s catalyst and the hydride-terminated (dimethylsiloxane) cross-linker were used as a stock solution. Karstedt’s catalyst stock solution was prepared by adding 5.8 μL of Karstedt’s catalyst solution in 2 mL of toluene. The cross-linker stock solution was prepared by dissolving 45 μL of the cross-linker in 400 μL of toluene. For 100 mg of PDES, 50 μL of Karstedt’s catalyst stock solution and 100 μL of toluene were added. After stirring the mixture, the desired amount of the cross-linker stock solution was added and stirred using a Thinky Mixer for 3 min. The mixture was cast on the glass slide coated with Rain-X and then thermally cross-linked at 65 °C for 48 h to ensure complete cross-linking. During cross-linking, the top surface was left exposed so that toluene could evaporate during the process.

### 4.4 Synthesis of the Twisting Actuator

The twisting actuator was prepared by attaching the PDMS elastomer onto the prestretched PDES elastomer. 60% cross-linked PDES elastomers were cut into dimensions of 1.3 × 3 × 0.21 mm^3^. The PDES elastomer was stretched by 250% at room temperature, with both ends of the actuator taped. On the top of PDES, the PDMS elastomer was attached using Ecoflex 00–35 Fast. The PDMS elastomer was made with Sylgard 184 at a weight ratio of 10:1 base to cross-linker. After curing of Ecoflex 00–30 Fast at room temperature, tapes were removed, resulting in a twisted actuator. For the actuation test, the twisting actuator was slightly stretched by a load (12 g). The actuator was first cooled to 12 °C inside the fridge and then heated to 40 °C using a heat lamp. The cycle was repeated multiple times to evaluate the reversibility of the actuation.

### 4.5 Synthesis of Controls (Neat PDMS, Sylgard 184, Ecoflex 00–30, LCEs, and SMPs)

Neat PDMS elastomers were synthesized via hydrosilylation. Linear PDMS copolymers containing vinyl side groups (200 mg) were mixed with the hydride-terminated PDMS cross-linker and Karstedt’s catalyst stock solution (100 μL). Karstedt’s catalyst stock solution was prepared by adding 5.8 μL of Karstedt’s catalyst solution in 2 mL of toluene. The PDMS cross-linker was added at 6 and 9 wt % for synthesizing neat PDMS elastomers with low and high cross-link densities, respectively. After stirring using a Thinky Mixer for 2 min, the mixture was cast on a glass slide and thermally cross-linked at 65 °C for 48 h to ensure complete cross-linking.

PDMS elastomers based on Sylgard 184 were prepared by mixing the Sylgard 184 base and curing agent. The weight ratio between the base and curing agent was set at 20:1 and 30:1 for preparing PDES elastomers with high and low cross-link densities, respectively. After degassing the mixture in a desiccator under vacuum, the mixture was cast on a glass slide and thermally cross-linked at 110 °C. PDMS elastomers based on Ecoflex 00–30 were prepared by mixing the Ecoflex 00–30 base and curing agent at a weight ratio of 1:1. After degassing the mixture in a desiccator under vacuum, the mixture was cast on a glass slide and cross-linked at room temperature.

LCEs were synthesized by two-step reactions. First, Michael addition of acrylate mesogens and thiol chain extenders was carried out to synthesize the liquid crystal oligomer. RM82, EDDT, and TATATO were mixed in a molar ratio of 0.8 (RM82):1 (EDDT):0.2 (TATATO). The mixture was combined with 2 wt % BHT, 3.5 wt % photoinitiator with a ratio of 0.25 (I-819):0.75 (I-369), and 1 wt % TEA. The mixture was stirred and oligomerized for 3 h at 65 °C. Following the first reaction, the oligomer was sandwiched between glass slides coated with Rain-X. The oligomer pressed between glass slides was heated and cooled 3 times to remove the alignment. The oligomer was photo-cross-linked at room temperature under 365 nm ultraviolet (UV) light under an intensity of 10 mW/cm^2^ (Excelitas Technologies, OmniCure LX500 UV LED) for 10 min per side to produce the polydomain LCE film.

Thermoresponsive, cross-linked PCL-DA (*M*_n_–10 kg mol^–1^) films were chosen to serve as SMPs. PCL-DA was synthesized by acrylation of PCL-diol in accordance with a prior report.^66^ Films were prepared by dissolving PCL in dichloromethane (0.15 g mL^–1^). The photoinitiator solution (10 wt % DMP in NVP) was then added at 15 vol %, mixed atop a shaker plate (1 min, 200 rpm), and pipetted into a silicone mold (*d*–50 mm x *t*–2 mm, McMaster-Carr) secured between glass slides. The mold was then cross-linked over a UV plate (UV-Transilluminator, 6 mW cm^–2^, 365 nm) for 5 min, flipping halfway through. The films were dried under vacuum (5 h, 30 in. Hg, room temperature) and then annealed at 85 °C (1 h). After resting for 5 h, the films were cut with a razor blade into their final dimensions (3 × 3 cm^2^).

### 4.6 Thermal Characterization

Differential scanning calorimetry (DSC) was performed using a TA Instruments DSC 2500 differential scanning calorimeter. A sample of approximately 10 mg was loaded into an aluminum hermetic DSC pan. The sample was first cooled to −90 °C and then heated to 100 °C. The temperature ramp was repeated for three cycles at a rate of 10 °C/min. Thermal analysis was repeated 3 times (*n* = 3).

### 4.7 Mechanical Characterization

Mechanical analysis of materials was carried out by a dynamic mechanical analyzer (DMA) (TA Instruments, RSA-G2). For quasi-static tensile testing, rectangular strips of PDES elastomers with dimensions approximately 2.5 × 7.5 × 0.4 mm^3^ were loaded and stretched at a constant linear rate of 0.13 mm/s. To measure the blocking stress, the sample was first stretched by 250% at room temperature. Under a constant strain of 250%, the temperature was ramped from room temperature to 0 °C at a rate of 3 °C/min. After the mixture was cooled to 0 °C, the temperature was ramped to 40 °C. This process was repeated to measure the cyclic blocking stress. For actuation strain measurements, the sample was first stretched by 250% at room temperature, and then the temperature was cooled to 0 °C. The force at 0 °C was maintained, and the sample was heated to 40 °C at a rate of 3 °C/min. This process was repeated to measure the cyclic actuation strain. For dynamic mechanical analysis, PDES elastomers of dimensions approximately 4.85 × 15.8 × 0.45 mm^3^ were loaded and tested in tensile mode at 1.5% strain at 0.25 Hz and heated from −40 to 30 °C at a rate of 3 °C/min. All tests were repeated 3 times for each composition (*n* = 3).

### 4.8 Gel Fraction

PDES films were weighed to obtain an initial mass (*M*_initial_). In a 20 mL vial, each sample was immersed in chloroform for 72 h at room temperature. The solvent was extracted by first air-drying the samples for 24 h and then vacuum-drying at 60 °C for 48 h until a final mass (*M*_final_) was achieved. The gel fraction was obtained using the following final equation

$$gel\;fraction\;\left(\%\right)=\frac{M_{final}\left(mg\right)}{M_{initial}\left(mg\right)}\times 100$$

All gel fraction tests were performed 3 times for each composition (*n* = 3).

### 4.9 Degradation Tests

Samples were tested for degradation under accelerated oxidative and hydrolytic conditions. For an accelerated oxidative study, the solution was prepared by mixing 325 mg of CoCl_2_ in 10 mL of RO water, while 50 mL of 30% w/v H_2_O_2_ was poured in 15 mL of RO water in a separate beaker. The diluted H_2_O_2_ was then poured in with the CoCl_2_ solution. For an accelerated hydrolytic study, a 0.1 M NaOH solution was used in accordance with ASTM F1635. The solution was prepared by dissolving 400 mg of NaOH in 100 mL of RO water. PDES elastomers, LCEs, and SMPs were submerged in the media in sealed vials and placed in an oven at 37 °C. Media were refreshed every 3 days. For degradation studies of PDES elastomers (Figure 6a,6b), samples were removed after designated time points (3, 6, 9, 12, and 15 days) and blotted dry, and the mass was measured. The mass change was calculated as follows

$$mass\;change\;\left(\%\right)=\frac{M_{final}-M_{initial}}{M_{initial}}\times 100$$

where *M*_final_ is the final mass after the sample is patted dry and *M*_initial_ is the initial dry mass. Samples were returned to the medium after measurement. A comparison of the environmental stability of PDES elastomers, LCEs, and SMPs was performed by measuring the change in dried mass before and after degradation (Figure 6d). Samples were removed from the accelerative oxidative media after 15 days and dried in a vacuum chamber for 3 days. The final dried mass was compared to the initial mass to evaluate the extent of degradation for each material.

### 4.10 Gel Permeation Chromatography

Molecular weights of PDES polymers were determined by gel permeation chromatography (GPC). Samples were dissolved in tetrahydrofuran for 24 h at room temperature with a concentration of 3 mg/mL and then analyzed in a TOSOH Ambient Temperature GPC system with a 0.1 mL/min flow rate.

### 4.11 NMR

Molecular structures (including the molar fraction of the vinyl content) were determined with ^1^H NMR spectroscopy (Avance Neo 400 MHz spectrometer) with CDCl_3_ as the standard. The molar ratio of each repeat unit in the PDES copolymer was determined by the proportion of the integral area of (*c + d + e*)/*b*. The calculated molar ratio was compared to the number-average molecular weight measured by GPC to determine the number of repeat units in PDES copolymers.

### 4.12 WAXS

WAXS was collected using a Xeuss 3.0 system from Xenocs (Soft Matter Facility, Texas A&M University). The sample temperature and strain were controlled using a Modular Force Stage system (MFS-350 with a 2N load cell, Linkam) connected to a T96-S controller (Linkam). The incident X-ray wavelength was λ = 1.54189 Å. Full Debye–Scherrer rings were integrated over 120 s intervals on a DECTRIS Si 1 M Eiger photon-counting area detector at different temperatures and tensile strains.

Self-developed MATLAB-based scripts leaning on concepts from a previous report^67^ were used to analyze the stored images. The data analysis relies on the image processing of the recorded diffracted patterns. To reduce noise and artifacts from air scattering, the background intensity, taken at the exact same experimental conditions without the presence of the sample, was subtracted from the recorded patterns. To calculate the scattered intensity of each reflection, we rebinned the two-dimensional (2D) detector images from orthogonal to polar spaces using azimuthal integration intervals of dϕ = 1° and radial intervals of d*q* = 0.005 Å^–1^. Additional noise reduction was achieved by averaging the signal over two 10*°* intervals around the fiber axis (meridian) for evaluating peaks along the scattering vector, *q*, and a 0.04 Å^–1^ interval for evaluating intensity distributions along the azimuthal angle, ϕ.

Quantitative analysis of the emerging mesophase peak intensity was performed by fitting Voigt profiles to the averaged peaks after subtraction of the scattering profile at 0% strain, in which the sample is completely isotropic. This was performed by using the MATLAB Voigt model fit subroutine.^68^ The fitted peaks were then integrated to obtain the intensity values.

A Hermann’s order parameter was calculated by constructing an orientation distribution function (ODF), *f*(β)·sinβ, β∈[0, π], where β is the azimuthal angle, for each scattering image following the subtraction of the isotropic background. The procedure followed the Leadbetter–Norris methodology as outlined in previous reports.^69–71^ Briefly, the full 360° azimuthal intensity profile was fitted using spherical harmonics in the form of a series of Legendre polynomials of even degrees

$${I\left(\phi ,q\right)=\Sigma }_{n=0}^{\infty }f_{2n}\frac{\left(2^{n}n!\right)}{\left(2n+1\right)!!}cos^{2n}\phi$$

where *f*_2n_ are the fitting parameters (Figure S5). The fitted parameters were then readily used to construct an angular distribution function (Figure S5) as a series of even-powered cosines

$${f\left(\beta \right)=\Sigma }_{n=0}^{\infty }f_{2n}\mathrm{co}s^{2n}\phi$$

The Hermann’s order parameter is then the normalized amplitude of the second-order spherical harmonics

$$S=\frac{1}{2}\left(3\left\langle \cos^{2}\phi \right\rangle -1\right)$$

where

$$\left\langle \cos^{2}\phi \right\rangle =\frac{\int_{0}^{\pi /2}f\left(\beta \right)\cos^{2}\beta \sin\beta d\beta }{\int_{0}^{\pi /2}f\left(\beta \right)\sin\beta d\beta }$$

## Supplementary Material







## Notes

Mechanical properties of silicone elastomers (Table S1); loading and unloading curves of 60% cross-linked PDES elastomers at different temperatures (Figure S1); loading and unloading curves of 40% cross-linked PDES elastomers at different temperatures (Figure S2); intensity and orientation of 110 reflections (Figure S3); effect of uniaxial stretching on the scattered intensity at the 111 reflection modulus (Figure S4); and effect of uniaxial stretching on the scattered intensity at the 110 reflection modulus on the orientation distribution functions (Figure S5) (PDF)
Phase transition from the aligned mesophase to the amorphous phase during unloading of the PDES elastomer (Supporting Video 1) (MP4)
Thermally responsive twisting and untwisting of the PDES bilayer actuator (Supporting Video 2) (MP4)
