# Supplementary material for: Phase-Change Silicone Elastomers for Tough, Soft Actuators
Source: Macromolecules. 2025 Jul 31;58(15):8067–78. doi: 10.1021/acs.macromol.5c01221 (PMC12356075; doi:10.1021/acs.macromol.5c01221)
Supplement: Supplementary file 1 [file ma5c01221_si_001.pdf]

Supporting Information

# Phase Change Silicone Elastomers for Tough, Soft Actuators

*Yoo Jin Lee*[1], *Asaf Dana*[1,2], *Sasha M. George*[2], *Manivannan Sivaperuman Kalairaj*[1], *Yeh-Chia Tseng*[1], *Brandon M. Nitschke*[1], *Jared A. Gibson*[1], *Melissa A. Grunlan*[1,2,3], *Taylor H. Ware*[1,2*]

**Table S1.** Mechanical properties of silicone elastomers.
**Figure S1.** Loading and unloading curves of 60 % crosslinked PDES elastomers at different temperatures.
**Figure S2.** Loading and unloading curves of 40 % crosslinked PDES elastomers at different temperatures.
**Figure S3.** Intensity and orientation of 110 reflections.
**Figure S4.** Effect of uniaxial stretching on scattered intensity at 111 reflection modulus.
**Figure S5.** Effect of uniaxial stretching on scattered intensity at 110 reflection modulus on the orientation distribution functions.

**Supplementary Video 1.** Phase transition from aligned mesophase to amorphous phase during unloading of PDES elastomer. 60 % crosslinked PDES elastomer was first uniaxially stretched by 320 % over its initial length at 10 °C to form mesophase. When unloaded, the elastomer undergoes phase transition from aligned mesophase into amorphous phase, accompanied by renecking. During the process, aligned mesophase and amorphous phase coexist, and the progression toward fully amorphous phase is visually captured. 1, 2, and 3 correspond to the steps marked in Figure 3d and 3e.

**Supplementary Video 2.** Thermally-responsive twisting and untwisting of PDES bilayer actuator. The bilayer actuator consists of stretched PDES as an active layer and unstretched PDMS as a passive layer. Prior to actuation, the actuator is cooled to 12 °C to induce mesophase formation in PDES. Upon heating to 40 °C, PDES contracts along its long axis due to phase transition, while PDMS remains nearly unchanged. The difference in the length of PDES and PDMS causes the actuator to twist. When cooled, PDES expands. The length of PDES and PDMS becomes better matched, resulting in untwisting of the actuator. The video is shown at 25× faster than true speed.

| | Young's modulus (kPa) | Failure strength (MPa) | Failure strain (%) | Toughness (MJ $m^{-3}$) |
|---|---|---|---|---|
| 40 % crosslinked PDES | 113.28 ± 15.50 | 0.31 ± 0.039 | 399.23 ± 27.46 | 0.55 ± 0.044 |
| 60 % crosslinked PDES | 227.32 ± 3.86 | 0.60 ± 0.10 | 395.81 ± 23.80 | 1.00 ± 0.14 |
| 80 % crosslinked PDES | 370.48 ± 26.11 | 0.73 ± 0.10 | 367.89 ± 24.88 | 1.35 ± 0.19 |
| Neat PDMS (Lower XL density) | 209.80 ± 7.61 | 0.14 ±0.034 | 103.63 ± 40.63 | 0.081 ± 0.038 |
| Neat PDMS (Higher XL density) | 353.80 ± 13.62 | 0.26 ± 0.085 | 92.08 ± 26.71 | 0.17 ± 0.10 |
| Sylgard 184 (Lower XL density) | 148.50 ± 12.18 | 0.33 ± 0.035 | 235.68 ± 15.42 | 0.33 ± 0.032 |
| Sylgard 184 (Higher XL density) | 461.73 ± 28.54 | 0.48 ± 0.084 | 133.05 ± 16.94 | 0.32 ± 0.076 |
| Ecoflex 00-30 | 69.51 ± 3.91 | 0.62 ± 0.028 | 683.27 ± 37.04 | 1.66 ± 0.19 |

**Table S1.** Mechanical properties of silicone elastomers. All values were measured three times for each composition ($n$ = 3).

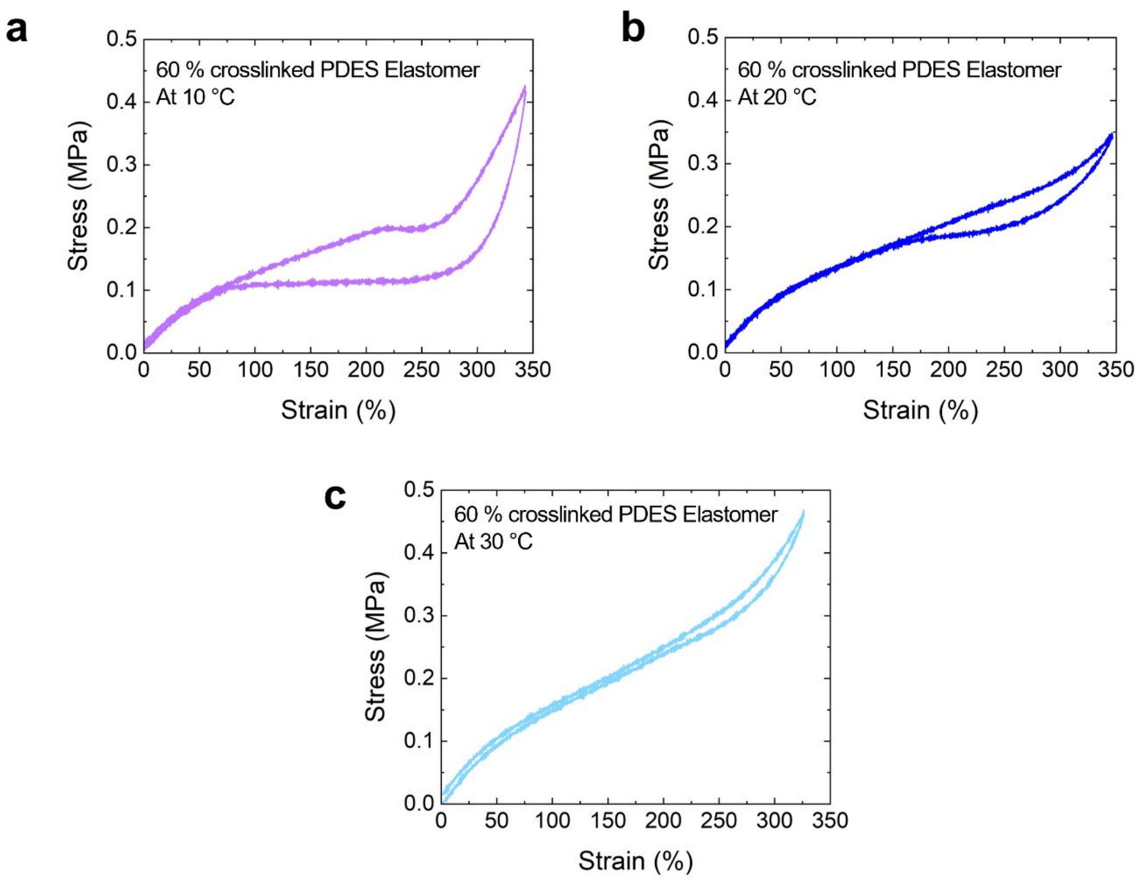


**Figure S1.** Loading and unloading curves of 60 % crosslinked PDES elastomers at different temperatures. 60 % crosslinked PDES elastomers were uniaxially stretched to ~ 350 % and then unloaded at 10 °C (a), 20 °C (b), and 30 °C (c).

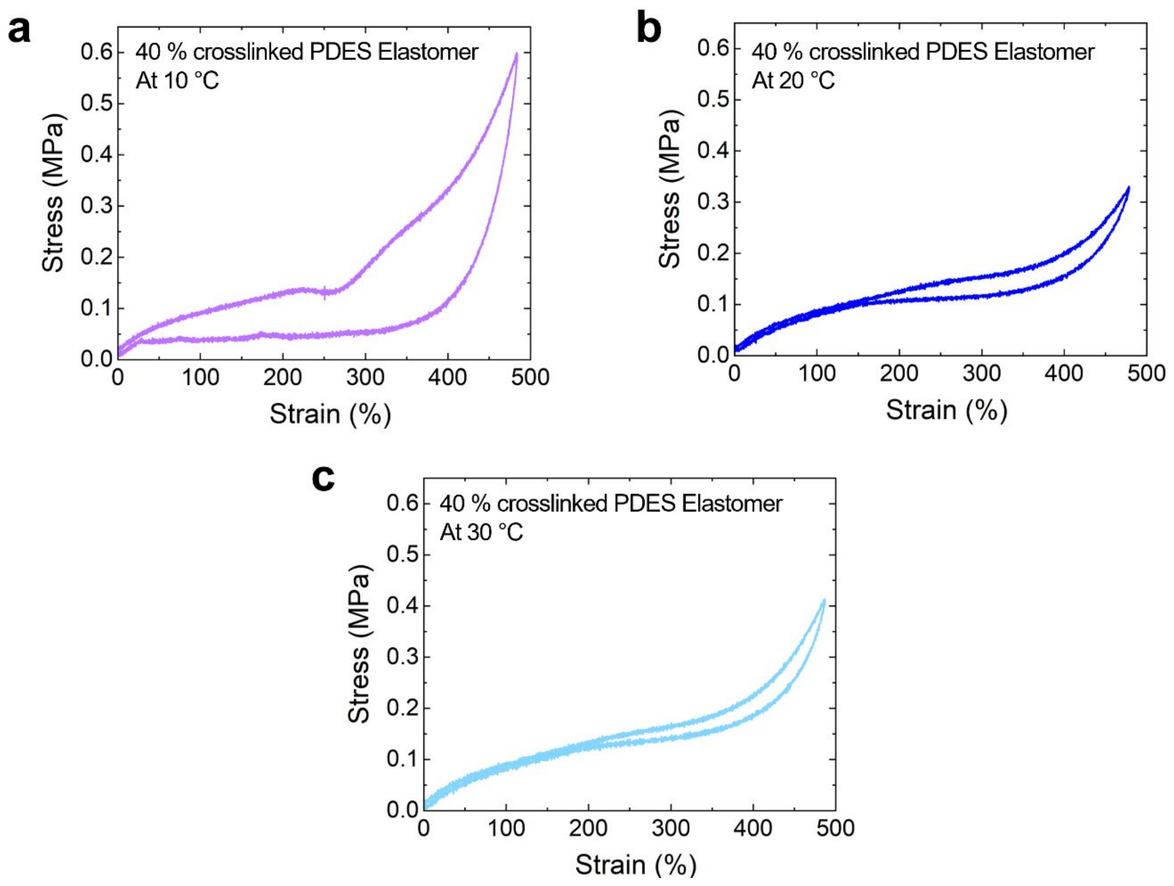


**Figure S2.** Loading and unloading curves of 40 % crosslinked PDES elastomers at different temperatures. 40 % crosslinked PDES elastomers were uniaxially stretched to ~ 500 % and then unloaded at 10 °C (a), 20 °C (b), and 30 °C (c).

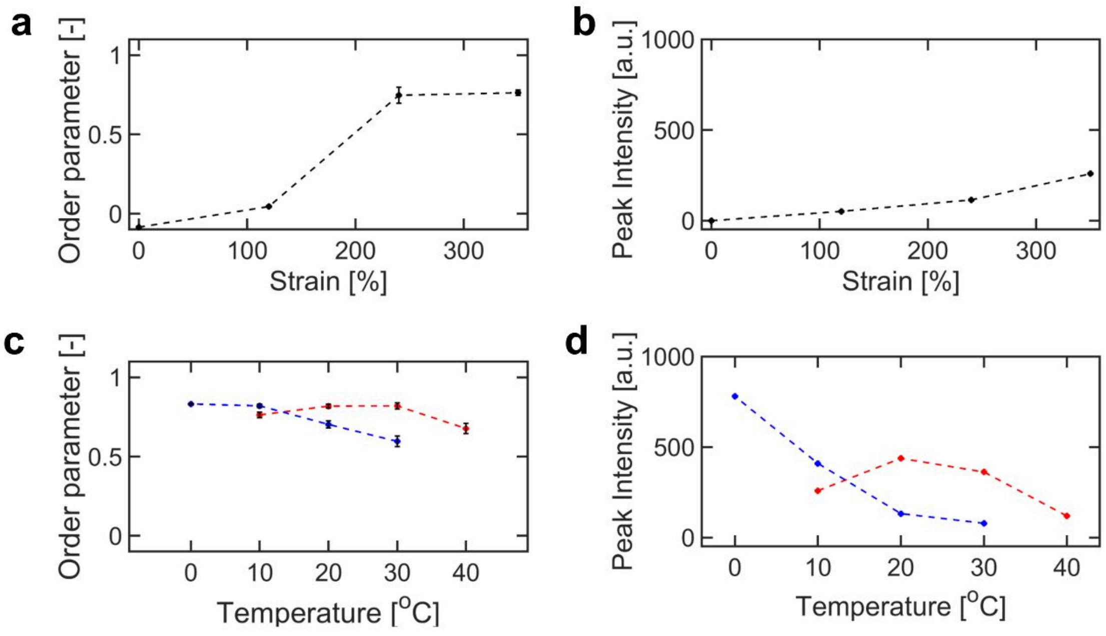


**Figure S3.** Intensity and orientation of 110 reflections. Orientational order parameter (a) and integrated 110 peak intensity (b) at a fixed temperature 10 °C taken at different values of uniaxial strain. Orientational order parameter (c) and integrated 110 peak intensity (d) at a fixed uniaxial strain (ε = 350 %) taken at different temperatures during the first heating (red markers) and cooling (blue markers) cycle immediately following sample stretching. Error bars represent averaging over the four quadrants.

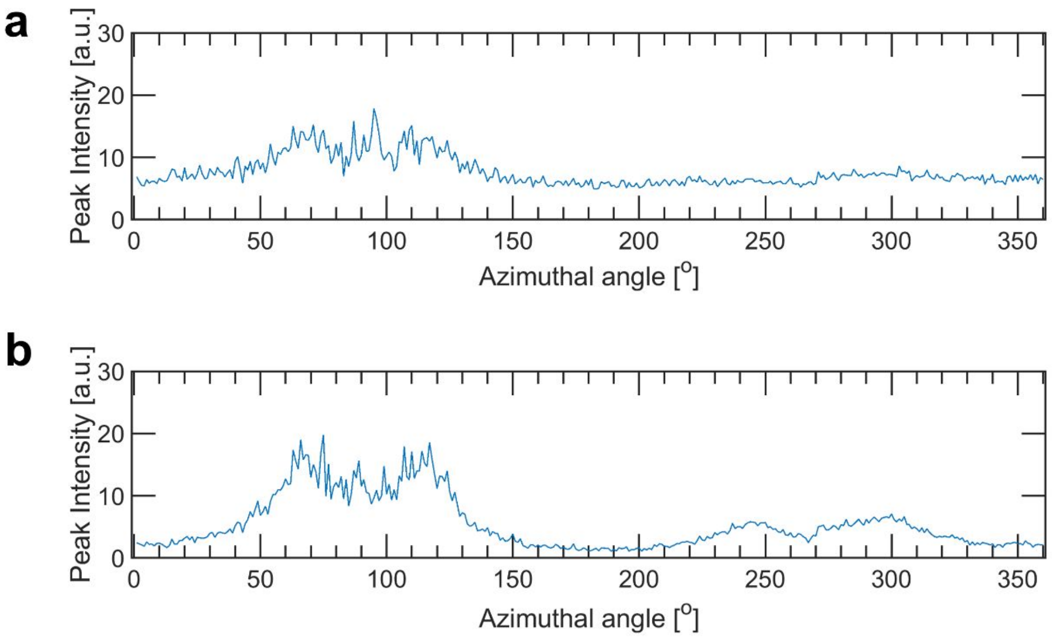


**Figure S4.** Effect of uniaxial stretching on scattered intensity at 111 reflection modulus. Scattered intensity at the 111 reflection modulus ($q$ = 1.55 Å$^{-1}$) as a function of the azimuthal angle for (a) unstretched sample ($\varepsilon$ = 0) at 10 °C and (b) stretched sample ($\varepsilon$ = 350 %) at 0 °C. When the sample is stretched and cooled, an increase in anisotropy is observed as the amorphous ring disappears and the 111 reflections appear at angles near the equatorial (i.e., 90 ° and 270 °.

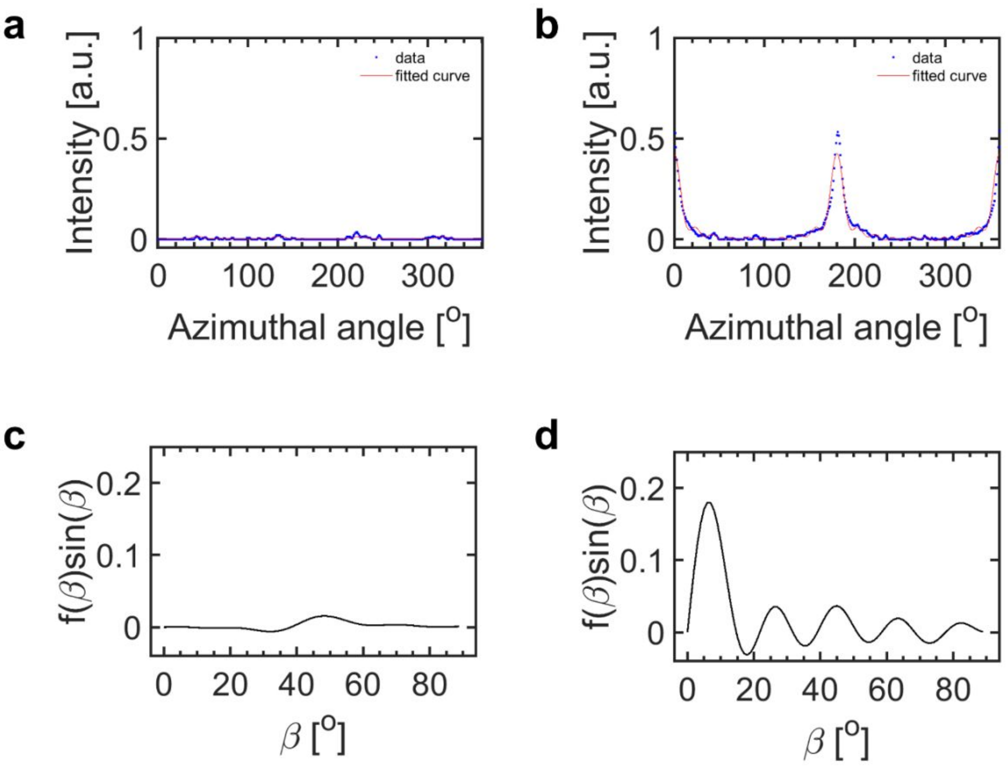


**Figure S5.** Effect of uniaxial stretching on scattered intensity at 110 reflection modulus on the orientation distribution functions. Raw (blue markers) and fitted profile (red curve) of the scattered intensity at the 110 reflection modulus ($q$ = 0.08 Å$^{-1}$) as a function of the azimuthal angle at 10 °C for (a) unstretched sample (ε = 0 %) and (b) stretched sample (ε = 350 %). The curves in (c) and (d) are representative orientation distribution functions f(β)sin(β), where β is azimuthal angle, constructed from a single quadrant of the fitted curve in (a) and (b), respectively.
